# The intra-tumoural stroma in patients with breast cancer increases with age

**DOI:** 10.1007/s10549-019-05422-6

**Published:** 2019-09-18

**Authors:** Kiki M. H. Vangangelt, Claire J. H. Kramer, Esther Bastiaannet, Hein Putter, Danielle Cohen, Gabi W. van Pelt, Emad A. Rakha, Andrew R. Green, Rob A. E. M. Tollenaar, Wilma E. Mesker

**Affiliations:** 1 Department of Surgery, Leiden University Medical Center, Albinusdreef 2, 2333 ZA Leiden, The Netherlands; 2 Department of Pathology, Leiden University Medical Center, Leiden, The Netherlands; 3 Department of Biomedical Data Sciences, Leiden University Medical Center, Leiden, The Netherlands; 4 Division of Cancer and Stem Cells, Nottingham Breast Cancer Research Centre, School of Medicine, Nottingham City Hospital, The University of Nottingham, Nottingham, UK

**Keywords:** Breast cancer, Tumour-stroma ratio, Ageing, Microenvironment, Prognosis

## Abstract

**Purpose:**

The tumour microenvironment in older patients is subject to changes. The tumour–stroma ratio (TSR) was evaluated in order to estimate the amount of intra-tumoural stroma and to evaluate the prognostic value of the TSR in older patients with breast cancer (≥ 70 years).

**Methods:**

Two retrospective cohorts, the FOCUS study (*N* = 619) and the Nottingham Breast Cancer series (*N* = 1793), were used for assessment of the TSR on haematoxylin and eosin stained tissue slides.

**Results:**

The intra-tumoural stroma increases with age in the FOCUS study and the Nottingham Breast Cancer series (*B* 0.031, 95% CI 0.006–0.057, *p* = 0.016 and *B* 0.034, 95% CI 0.015–0.054, *p* < 0.001, respectively). Fifty-one per cent of the patients from the Nottingham Breast Cancer series < 40 years had a stroma-high tumour compared to 73% of the patients of ≥ 90 years from the FOCUS study. The TSR did not validate as an independent prognostic parameter in patients ≥ 70 years.

**Conclusions:**

The intra-tumoural stroma increases with age. This might be the result of an activated tumour microenvironment. The TSR did not validate as an independent prognostic parameter in patients ≥ 70 years in contrast to young women with breast cancer as published previously.

## Introduction

Breast cancer is the leading malignancy in European women [1]. A major risk factor for breast cancer development is ageing [2].

In the last decade, the tumour microenvironment has gained interest in unravelling cancer development and cancer progression, but also as a source for new therapeutic targets and prognostic parameters. The tumour microenvironment, i.e. tumour stroma, consists of a variety of structures and cells located in the extracellular matrix, such as immune cells, fibroblasts and endothelial cells. Various processes in the tumour microenvironment are involved in tumour progression by influencing the proliferation of cancer cells, the epithelial–mesenchymal transition, tumour metabolism and dissemination capabilities [3]. Epidemiological and clinico-pathological characteristics are different in older patients with breast cancer compared to their younger counterparts [4–7]. The biology of breast cancer is age dependent in which alterations in extracellular matrix and products secreted by senescent fibroblasts are thought to promote late-onset breast tumourigenesis; however, the extent is still unknown [8]. Research into the molecular profile of older patients with triple negative breast cancer showed a different stromal microenvironment favourable for tumourigenesis, in which senescence-associated secretory profile and autophagy are important aberrant stromal features induced with increasing age [9].

A widely researched prognostic marker based on the tumour-microenvironment is the tumour–stroma ratio (TSR). The TSR reflects the ratio between tumour cells and stromal cells and is visually assessed with conventional light microscopy. Previous studies have shown that the TSR is a valuable prognosticator for breast cancer patients, whereby tumours with a high-stromal content are associated with a poor clinical outcome [10–18]. This effect was observed and validated in the overall group of breast cancer patients and clinically relevant subgroups [18].

In the current literature, older patients are often defined as patients of 70 years and older [19]. In older patients with breast cancer, better risk stratification is desirable. Whilst breast cancer mortality in the total group of patients with breast cancer has decreased over the last decade, this decrease is lower or absent in older patients. This leads to an increased survival gap between older and younger patients with breast cancer [20–23]. Invasive breast tumours in the ageing women are thought to have a more favourable biology compared to younger females. Improvement of prognostic tools is needed for more accurate prediction of prognosis in the older breast cancer patient, considering that only very few older patients with breast cancer aged over 70 years receive chemotherapy [24]. More accurate stratification of disease aggressiveness could contribute in shared-decision making on the extent of adjuvant therapy. This may minimise the risk of undertreatment which may contribute in the survival gap between younger and older patients with breast cancer. Although extensive research in population-based studies showed that the TSR is an important prognosticator in women with breast cancer, none of these studies have focused on its significance in the older female population.

Therefore, the aims of this study were (1) to investigate the amount of intra-tumoural stroma by the assessment of the TSR in older patients with breast cancer and (2) to evaluate the prognostic value of the TSR in women diagnosed with breast cancer at the age of 70 years or older.

## Materials and methods

### Study population

This study included two databases with retrospectively collected clinical data from women diagnosed with breast cancer.

#### The FOCUS study

The FOCUS study consisted of a population based cohort of women aged 65 years and older, who were diagnosed with breast cancer (*N* = 3672) between 1997 and 2004 in Comprehensive Cancer Centre Region West (the Netherlands). Women with a history of cancer or in situ tumours, neoadjuvant therapy, distant metastasis at time of diagnosis, age under 70 years or with no available tumour tissue were excluded. In total, 1577 women were suitable for analyses. This cohort was used to answer both study aims, the evaluation of the amount of intra-tumoural stroma and the prognostic value of the TSR in the older women with breast cancer.

#### The Nottingham Breast Cancer series

The Nottingham Breast Cancer series (*N *= 1809) is a cohort of women ≤ 70 years of age presenting with primary invasive breast cancer without distant metastasis, and primary treated with surgery in Nottingham City Hospital between 1993 and 2002. Patients were included if haematoxylin and eosin (H&E) stained tissue slides and clinical information (patients and tumour characteristics and survival data) were available. This study was used for the evaluation of the amount of intra-tumoural stroma with the increase of age.

For standard clinical care all resected tumours were assessed by a pathologist, according to the currently applied pathological standards. The clinical data from the Nottingham Breast Cancer series were anonymized and the study was approved by the Nottingham Research Ethics Committee 2 under the title ‘Development of a molecular genetic classification of breast cancer’. All samples from the FOCUS study were also anonymised and data were handled according to national ethical guidelines (“Code for Proper Secondary Use of Human Tissue”, Dutch Federation of Medical Scientific Societies).

### Tumour–stroma ratio assessment

**Fig. 1:**
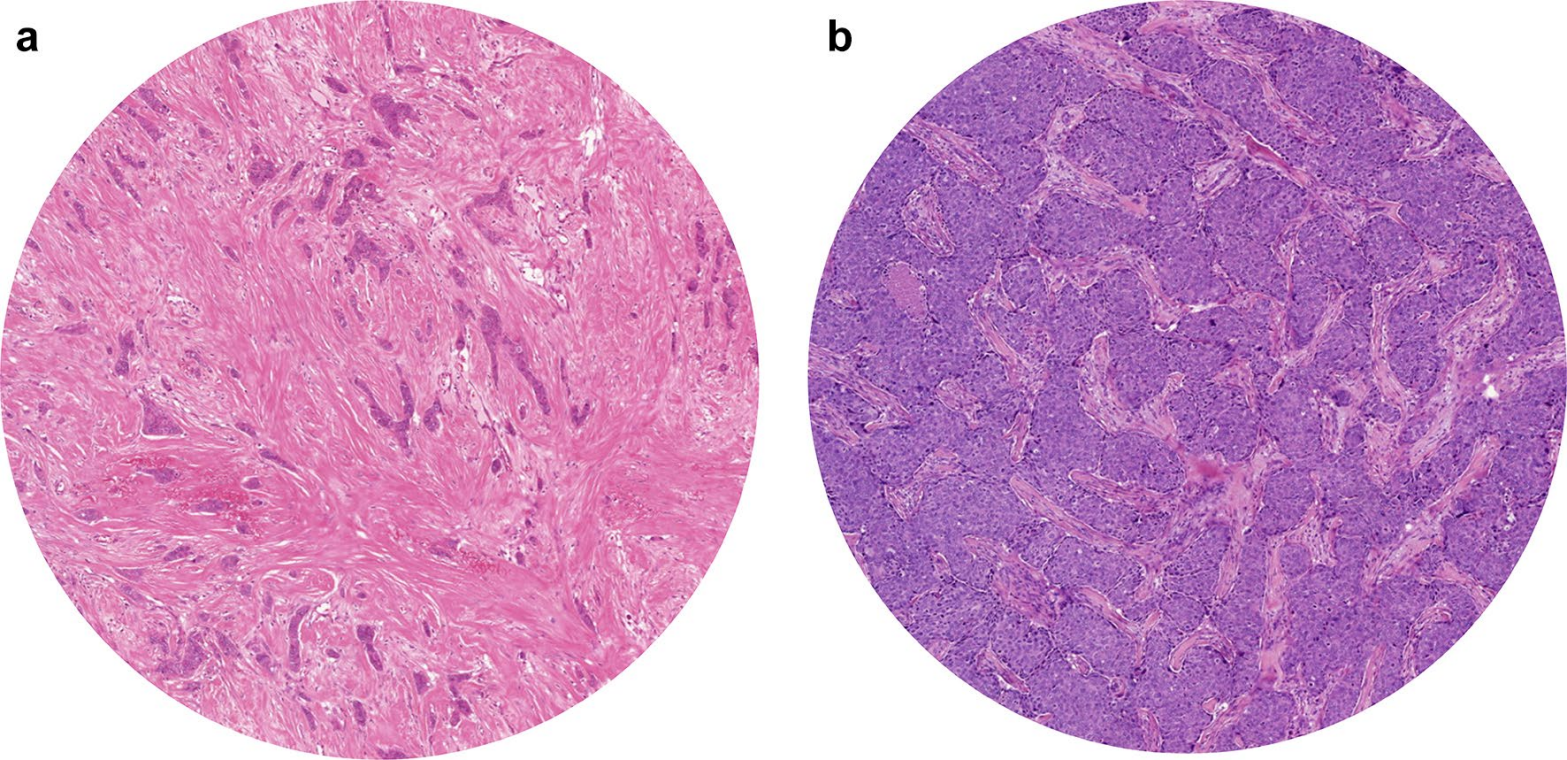
Representative example of tumour–stroma ratio assessment **a** stroma-high tumour, **b** stroma-low tumour

The tissues slides from the FOCUS study were assessed for the TSR by visual eyeballing with a conventional light microscope on standard H&E stained tissue slides, as described previously by our group [10, 25]. The most stroma rich area on the slide was selected with a 5× objective. A 10× objective was used to select the final most stroma abundant area. The H&E slides from the Nottingham Breast Cancer series were digital assessed via CaseViewer 2.2 for windows (3D HISTECH Ltd.). The original H&E slides were scanned with a 20× magnification using 3D Histech Panoramic 250 Flash II (3DHISTECH Ltd., Budapest, Hungary). Also for digital assessment the most stroma abundant area was selected. In the most stroma rich field a circle with an area of 3.1 mm^2^ was annotated. This area corresponded with the magnification used in our previous published research [26]. The next steps in the assessment of the TSR on digital images and conventional images were performed in the same manner. The percentage of stromal cells compared to tumour cells in the selected area were scored by increments of 10%. The selected area required tumour cells at all borders of the image field. Stromal areas with post-biopsy effects were avoided. Finally, the determined percentages were divided into two categories; stroma-low (≤ 50% stroma) and stroma-high (> 50% stroma) (Fig. 1). The tissues slides were double scored in a blinded fashion. If no consensus could be reached between the two observers a third observer was consulted. Consensus could be reached in all cases.

### Statistical analyses

For statistical analyses IBM statistics v23.0 (SPSS, Inc., an IBM Company Chicago, IL, USA) was used. Relative survival analyses were performed with STATA SE software version 12 (StataCorp, College Station, TX, USA). A Cohen’s Kappa was calculated for the evaluation of inter-observer agreement. A value above 0.6 was considered as good level of agreement. To evaluate the difference of patient characteristics between women with stroma-low or stroma-high tumours a *χ*^2^ test was used in case of categorical variables. The distribution of numerical variables was tested with the Shapiro–Wilk test. Non-parametric continuous variables were evaluated using Mann–Whitney *U* test. Linear regression analysis was performed to investigate the association between age (continue) and the intra-tumoural stroma in percentage (increments of 10%). The linear regression analyses was adjusted for tumour size, histology, oestrogen receptor (ER) status, progesterone receptor (PR) status, human epidermal growth factor 2 (HER2) status, triple negative (TN) status and grade, as these parameters might influence the amount of intra-tumoural stroma.

The primary endpoint was recurrence free period (RFP). The definition for RFP was time from diagnosis to local, regional or distant recurrence or contralateral breast cancer. Censoring was applied at the last date at which patients were known to be recurrence free and alive. The secondary endpoint was relative survival (RS). The observed overall survival (OS) among included patients divided by the expected survival in the sex-, age-, and calendar year matched general population was defined as relative survival. This was applied according to the Ederer II method with use of the ‘strs’ command in STATA. A relative survival rate of less than 100% at 10 years after diagnosis means that the survival of patients in the study is lower than expected when compared to survival of the general population. The relative survival data were calculated at 10 years of follow-up. The relative excess risk of death (RER) was estimated using a multivariable generalized linear model with a Poisson distribution, based on collapsed relative survival data, using exact survival times. To assess the differences in RFP for our parameter of interest, the Kaplan–Meier curves were compared using log-rank test. This test was also used for analysing different TSR cut-off values, other than the normally used 50% (i.e. ≤ 50% stroma is categorized as stroma-low and > 50% stroma is categorized as stroma-high). A *p* value lower than 0.05 was considered statistically significant for all analyses. Cox regression analyses were used to calculate the prognostic value of the TSR (univariate analysis and multivariate). The TSR was corrected for clinical important confounders. The interaction term was introduced to evaluate the prognostic value of the TSR stratified by confounders. Power analyses showed that at least 618 patients of the FOCUS study must be analysed to reach a power of 0.80 (1-β) with a type I error rate of 5% (α).

## Results

### Patients

#### The FOCUS study

In total, 1577 women included in the FOCUS study were eligible for inclusion. Based on power calculation, 627 patients were selected via computer randomisation (minimum of 618 patients). The included (*N *= 627) and excluded (*N* = 950) patients were compared for age, tumour grade, histological type, T-stage, N-stage, hormone receptor status, HER2 status, type of operation, radiotherapy, chemotherapy and hormonal therapy. Between these two groups, only hormonal therapy showed to be statistically significant different (*p* = 0.003). In the included group, more patients were treated with hormonal therapy. However, hormonal therapy has no association with outcome (HR 1.01, 95% CI 0.66–1.54, *p *= 0.975). The median age of the excluded patients was 78 and the median age of the included women was 79 at time of diagnosis. Eight slides were not suitable for TSR assessment due to poor quality of the staining.

The characteristics of the selected patients are described in Table 1. Cohen’s kappa inter-observer agreement was 0.77 (33% of slides were scored in a double-blinded fashion).

**Table 1:** Statistically significant difference between stroma-low and stroma-high tumours in the FOCUS study

	*N*	Stroma-low (%) (*N* = 204)	Stroma-high (%) (*N* = 415)	*p* value
Age (in years)				
	619	79 (mean)	80 (mean)	0.020
Grade				
I	82	31 (22.0)	51 (17.3)	0.126
II	198	69 (48.9)	129 (43.9)	
III	155	41 (29.1)	114 (38.8)	
Histological type				
Invasive carcinoma of NST	471	148 (72.5)	323 (77.8)	0.171
Lobular	65	28 (13.7)	37 (8.9)	
Other	83	28 (13.7)	55 (13.3)	
Tumour size				
pT1	254	96 (47.1)	158 (38.1)	0.014
pT2	286	92 (45.1)	194 (46.7)	
pT3/4	79	16 (7.8)	63 (15.2)	
Tumour involvement in the lymph nodes				
Negative	353	134 (66.3)	219 (54.2)	0.004
Positive	253	68 (33.7)	185 (45.8)	
ER status				
Negative	95	33 (18.9)	62 (16.9)	0.574
Positive	447	142 (81.1)	305 (83.1)	
PR status				
Negative	195	64 (38.8)	131 (37.8)	0.822
Positive	317	101 (61.2)	216 (62.2)	
HER2 status				
Negative	484	151 (76.3)	333 (82.0)	0.096
Positive	120	47 (23.7)	73 (18.0)	
Type of surgery				
BCS	181	68 (33.3)	113 (27.2)	0.117
MST	438	136 (66.7)	302 (72.8)	
Radiotherapy				
No	366	121 (59.3)	245 (59.0)	0.947
Yes	253	83 (40.7)	170 (41.0)	
Chemotherapy				
No	602	199 (97.5)	403 (97.1)	0.753
Yes	17	5 (2.5)	12 (2.9)	
Hormonal therapy				
No	303	112 (54.9)	191 (46.0)	0.038
Yes	316	92 (45.1)	224 (54.0)	

*NST* no special type, *ER* estrogen receptor, *PR* progesterone receptor, *HER2* human epidermal growth factor receptor 2, *MST* mastectomy, *BCS* breast conserving surgery. Missing values were excluded from this analyses

#### The Nottingham Breast Cancer series

An external cohort of primary breast cancer patients diagnosed in Nottingham City Hospital was used for the evaluation of the TSR in order to investigate alterations in the amount of intra-tumoural stroma. Due to bad quality of the tissue, 15 patients were excluded (0.8%), and one patient was excluded because clinical information regarding patients age was unknown. Finally, 1793 patients were used in the analyses. The mean age was 55. An overview of patient characteristics, tumour characteristics and treatment is shown in Table 2. All slides were assessed by two observers. If no consensus could be reached a third observer was consulted. Consensus was reached in all cases.

**Table 2:** Statistically significant difference between stroma-low and stroma-high tumours in the Nottingham Breast Cancer series

	*N*	Stroma-low (%) (*N* = 681)	Stroma-high (%) (*N* = 1113)	*p* value
Age (in years)				
	1793	54 (mean)	55 (mean)	0.003
Grade				
I	279	105 (15.4)	174 (15.6)	0.779
II	733	272 (40.0)	461 (41.5)	
III	780	303 (44.6)	477 (42.9)	
Histological type				
Invasive carcinoma of NST	1128	450 (66.1)	678 (61.0)	0.114
Lobular	155	53 (7.8)	102 (9.2)	
Tubular	275	90 (13.2)	185 (16.6)	
Others	235	88 (12.9)	147 (13.2)	
Tumour size				
T1	1146	505 (74.3)	641 (57.7)	< 0.001
T2	624	169 (24.9)	455 (41.0)	
T3	21	6 (0.9)	15 (1.4)	
Tumour involvement in lymph nodes				
Negative	1127	452 (66.6)	675 (60.8)	0.013
Positive	663	227 (33.4)	436 (39.2)	
ER status				
Negative	331	151 (22.2)	180 (16.2)	0.002
Positive	1462	530 (77.8)	932 (83.8)	
PR status				
Negative	708	282 (42.0)	426 (38.7)	0.168
Positive	1066	390 (58.0)	676 (61.3)	
HER2 status				
Negative	1572	594 (87.2)	978 (87.9)	0.650
Positive	221	87 (12.8)	134 (12.1)	

*NST* no special type, *ER* estrogen receptor, *PR* progesterone receptor, *HER2* human epidermal growth factor receptor 2. Missing values were excluded from this analyses

### Alterations in stromal amount with the increase of age

**Fig. 2:**
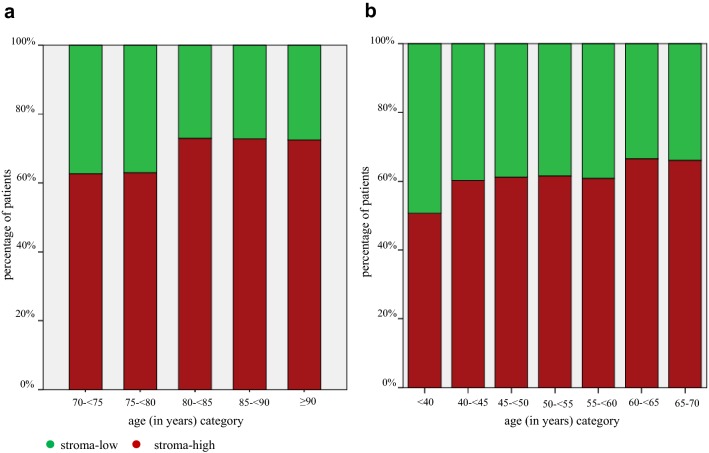
Percentage of patients with stroma-low and stroma-high tumours stratified by age category **a** the FOCUS study (*N* = 619), **b** the Nottingham Breast Cancer series (*N* = 1793)

For the patients in the FOCUS study (*N *= 619), the Mann–Whitney *U* test showed a significant association between age and TSR (*p* = 0.020). By evaluating the TSR, the results showed a higher amount of intra-tumoural stroma with the increase of age (*B* 0.025, 95% CI 0.004–0.045, *p* = 0.018). In the group of patients between 70 and < 75 years of age, 63% of the tumours were assessed as stroma-high compared to 73% of the tumours in patients aged 90 years or older (Fig. 2a).

To evaluate this age-effect in an independent cohort, the Nottingham Breast Cancer series (*N *= 1793) consisting of breast cancer patients of ≤ 70 years of age was assessed. The Mann–Whitney *U* test showed a significant association between age and TSR (*p *= 0.003). In this patient cohort, the evaluation of the TSR showed that the amount of intra-tumoural stroma also increases with age (*B* 0.033, 95% CI 0.014–0.053, *p* = 0.001). Of the patients under the age of 40, 51% were scored as stroma-high compared to 66% of patients between the 65 and 70 years of age (Fig. 2b).

Linear regression was adjusted for tumour size, histology, ER status, PR status, HER2 status, TN status and grade in the FOCUS study and the Nottingham Breast Cancer series (*B* 0.031, 95% CI 0.006–0.057, *p* = 0.016 and *B* 0.034, 95% CI 0.015–0.054, *p* < 0.001, respectively). These results showed that the association between the amount of intra-tumoural stroma and age remained statistically significant after adjustment of pathological tumour based characteristics.

### Evaluation of the prognostic value of the TSR older patients with breast cancer

#### The FOCUS study

**Fig. 3:**
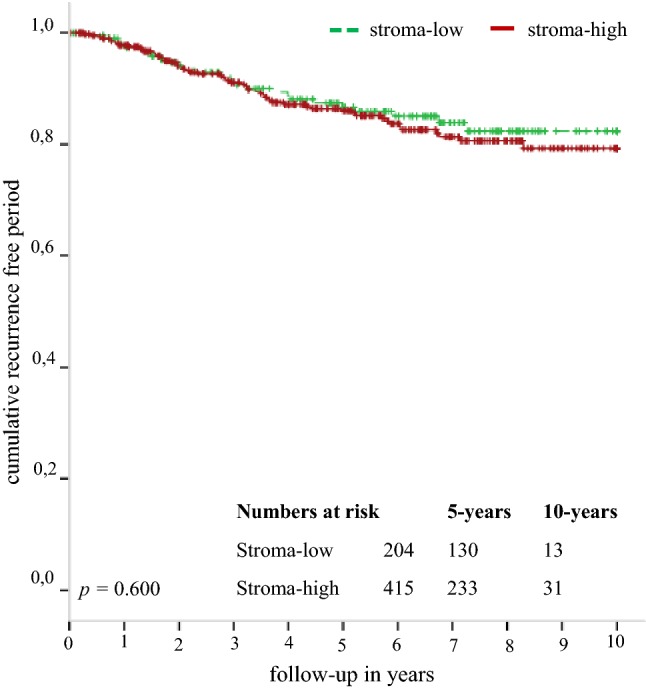
Kaplan–Meier analysis for recurrence free period stratified by tumour–stroma ratio of patients included in the FOCUS study

Most of the 619 tumours were categorised as stroma-high (67%). Eighty-five patients developed a tumour recurrence. Among stroma-high tumours, a higher number of patients with positive lymph nodes (*p* = 0.004), an advanced T-stage (*p* = 0.014) and hormonal therapy (*p* = 0.038) was observed. Older age was associated with stroma-high tumours (*p* = 0.020) (Table 1). After a follow-up period of 10 years no statistical significant differences were observed in recurrence rates between stroma-low and stroma-high tumours, 18% versus 21% respectively (HR 1.13, 95% CI 0.72–1.78, *p* = 0.602) (Fig. 3). The results in the multivariate Cox regression analysis were in line with the results of the univariate analysis (HR 1.02, 95% CI 0.59–1.78, *p *= 0.937) (Table 3). After 10 years of follow-up the relative survival rates of patients with stroma-low compared to stroma-high tumours were 90.2% versus 91.6%, respectively (RER 1.53, 95% CI 0.31–7.47, *p* = 0.601).

**Table 3:** Univariate and multivariate analyses for recurrence free period calculated by Cox regression analysis for the FOCUS study

	Univariate			Multivariate		
	HR	95% CI	*p* value	HR	95% CI	*p* value
Age	1.01	0.97–1.05	0.623	1.02	0.59–1.78	0.349
Grade						
I			< 0.001			< 0.001
II	1.28	0.52–3.17		1.29	0.47–3.53	
III	3.91	1.66–9.24		1.05	1.48–11.11	
Tumour size						
≤ 2 cm			0.003			0.053
> 2 cm	2.04	1.28–3.25		1.77	0.99–3.15	
Histological type						
Invasive carcinoma of NST			0.715			0.518
Lobular	0.82	0.39–1.70		0.57	0.17–1.87	
Others	0.80	0.41–1.55		1.26	0.59–2.69	
ER status						
Negative			< 0.001			0.735
Positive	0.39	0.25–0.63		1.13	0.56–2.26	
PR status						
Negative			0.008			0.004
Positive	0.54	0.35–0.85		0.41	0.22–0.75	
HER2 status						
Negative			0.158			0.637
Positive	1.43	0.87–2.34		0.86	0.47–1.60	
TSR						
Stroma-low			0.602			0.937
Stroma-high	1.13	0.72–1.78		1.02	0.59–1.78	

*NST* no special type, *HER2* human epidermal growth factor receptor 2, *PR* progesterone receptor, *ER* estrogen receptor, *TSR* tumour–stroma ratio

The interaction term was added in the Cox regression analyses. These analyses showed no statistical significant value for the TSR if stratified by grade (*p* = 0.571), morphology (*p* = 0.449), ER status (*p* = 0.598), PR status (*p* = 0.737), HER2 status (*p* = 0.721) or tumour size (*p* = 0.571).

In the FOCUS study, survival analyses were performed for the TSR at other cut-off values than the established 50%. The cut-off values ranged from 20% to 70% but none of the values showed statistically significant differences on clinical outcome (data not shown).

## Discussion

The results in this study showed a significant association between age and intra-tumoural stroma percentage expressed with the TSR; a higher amount of intra-tumoural stroma was observed with the increase of age. This may be related to differences in tumour development and tumour microenvironment in older patients with breast cancer compared to their younger counterparts. For instance due to age-related pathological alterations which occur in the mamma, such as an increase in fat tissue and collagenous stroma as replacement for glandular tissue [5, 27]. The extent of the alterations in the extracellular matrix and products secreted by senescent fibroblasts in the promotion of late-onset breast tumourigenesis is still unknown. A different view on the role of senescent cells is suggested in recent literature. Senescent cells were previously thought to be tumour-protective, but recent research showed that these cells contribute to a tumour-promoting environment [8]. A dysregulated response between declining immune function (i.e. immunosenescence) on one hand and a low grade chronic inflammation (i.e. inflammageing) on the other hand may lead to an altered tumour microenvironment that has impact on tumour development and tumour growth in the ageing population, probably with the involvement of CD4+ and CD8+ T-cells [28]. Previous research showed decreased values of these immune cells in mammary tumours in older mice compared to their younger counterparts [29]. Brouwers et al. investigated the molecular profile of the microenvironment in older triple negative breast cancer patients. The authors provided evidence that breast cancer in the older patients is associated with a different stromal microenvironment favourable for tumourigenesis, in which senescence-associated secretory profile and autophagy are important stromal features induced with age. As an illustration, the authors validated in an external publicly available dataset a significant upregulation of fibroblast growth factor 13 (FGF13) in tissues of older breast cancer patients. This gene belongs to the fibroblast growth factor superfamily. Aberrant expression of this superfamily is involved in tumour growth and invasion [9]. Another process that occurs with ageing are changes in the hormonal status. In postmenopausal women the production of oestradiol takes place in peripheral tissues instead of the production in the ovaries in the premenopausal status. This change leads to a consistent but lower level of circulating oestrogen [30]. Postmenopausal women with relatively high systemic concentration of oestrogen have a higher risk of developing breast cancer [31]. The chance of random genetic errors is increased by the proliferative effect of oestrogens on breast epithelial cells [32, 33]. Whether these processes contribute to the increase of stroma-high patients is not known yet. Also the contradictory results in this study regarding the prognostic value of the TSR is not fully understood. These results are in strong contrast to the discriminating power of the TSR regarding to clinical outcome presented in the review of Kramer et al. The authors showed that patients with stroma-high tumours have a poor clinical outcome. This was observed in the overall patient with breast cancer and in clinically relevant subgroups such as triple negative tumours, oestrogen positive tumours and lymph node negative tumours [18]. Therefore, understanding and confirming of age-related changes in the microenvironment requires further research.

Regarding the ageing patient, the tumours of older patients with breast cancer are for example more often receptor positive and have a lower grade [34]. In contrast to the more favourable biology, Van de Water et al. concluded that the clinical outcome in older patients with breast cancer must not be underestimated, as breast cancer relapse and disease specific mortality is higher in older breast cancer patients compared to their younger counterparts [35]. A study performed in Denmark showed results in line with Van de Water et al. The 5-year relative survival decreases with the increase of age; 90% for patients aged between 0 and 69 years, 80% for patients aged 70–79, 73% for women aged 80–89 years [22]. Also the frequently used online prediction tool PREDICT slightly overestimated the 10-years overall survival of patients aged ≥ 65 years and must especially be interpreted with caution in patients aged ≥ 75 years [36, 37]. Dutch guidelines contain no explicit recommendations about chemotherapy in the older patients, mainly due to the scarce amount of studies specifically focusing on older patients resulting in lack of evidence about the efficiency of chemotherapy in patients over 70 years. In daily clinical practice in the Netherlands, chemotherapy is advised in fit older patients over 70 years. Shared-decision making between oncologists and patients plays a role in this process. A better prediction rule for prognosis combined with research about the definition of ‘fit’, and the effectiveness and side effects of chemotherapy in older patients, might simplify decision making regarding adjuvant therapeutic options. Based on the result that TSR seems to be an important prognostic marker in patients under the age of 70 in contrast to older patients, we advocate for the importance of validating other prognostic parameters in older patients.

With respect to this study, the chosen endpoint might have an effect on the outcome of the prognostic value of the TSR. With RFP as primary endpoint, it remains possible that metastases or recurrences are not filed if the observation of disease relapse has no clinical consequence, for example if patients are unfit for further treatment. To minimise the effect of competing mortality on survival the second endpoint was determined as RS instead of OS. A final limitation of this study is that adjuvant treatment options have changed over the years. Advantages of the included studies are a long follow-up period and the amount of patients included in the FOCUS study. In order to give a more definitive conclusion about the prognostic value of the TSR in the older patient with breast cancer, a large observational population-based cohort study of older breast cancer patients treated following current guidelines assembled in a detailed database with focus on recurrences and disease specific survival is necessary.

## Conclusions

The intra-tumoural stroma increases with age. The TSR showed no correlation with survival in patients of 70 years or older in contrast to young women with breast cancer as published previously.

## Data Availability

Data are available on request.
